# Molecular Mechanisms of Venom Diversity

**DOI:** 10.3390/toxins17120581

**Published:** 2025-12-03

**Authors:** Marcela Akemi Ishihara, Adriana Rios Lopes, Milton Yutaka Nishiyama-Jr

**Affiliations:** 1 Laboratory of Applied Toxinology, Instituto Butantan, São Paulo 05585-000, SP, Brazil; 2 Laboratory of Biochemistry, Instituto Butantan, São Paulo 05585-000, SP, Brazil; adriana.lopes@butantan.gov.br

**Keywords:** venom, venomics, gene expression, phenotypic variation, drug discovery

## Abstract

Animal venoms are valuable resources for drug discovery. They offer a wide variety of bioactive molecules with significant biotechnological potential. Venom composition shows extensive diversity not only between and within species, but also across the lifetime of an individual. This natural variation further enhances the biotechnological potential of venoms, supporting the development and optimization of venom-derived drugs. Despite numerous studies highlighting the variability of venom, many lack a coherent framework to explain the underlying causes of this diversity. In this review, we explore the molecular and evolutionary mechanisms driving variations in venom composition and the evolution of venom systems, including gene regulation, point mutations, gene duplication events, modulation by miRNAs, alternative splicing and post-translational modifications as driving forces of venom component diversity. We also discuss the critical role of omics technologies and comparative studies in advancing our understanding of the diversity of venom and their contribution to the identification, development, and refinement of venom-based product candidates. The aspects reviewed here are relevant for future omics study designs to advance venom research and biodiscovery.

## 1. Introduction

Animal venoms provide a powerful model for studying how proteins and molecular pathways can be repurposed into novel biological functions throughout evolution. This raises fundamental questions about the molecular mechanisms that enable such adaptations and the emergence of potent biochemical repertoires from a relatively conserved molecular toolkit [1]. Despite progress, the molecular forces shaping venom diversity remain incompletely understood [2]. The independent origin of venom systems in more than 100 lineages further complicates this scenario, as distinct evolutionary pressures and mechanisms may underlie the origin and diversification of venom systems [3]. Moreover, venom composition is highly variable, not only among distantly related taxa but also between closely related species, between sexes within a species, and across developmental stages of a single individual [4,5,6].

Extensive venom variation can be attributed to ecological factors and adaptations. Venoms can be deployed for multiple ecological purposes. In the Animal Kingdom, animals use venom for predation, defense and, in some lineages, reproduction [7,8]. Variations in the venom delivery system apparatus are also observed among lineages [7]. Given the ecological relevance of animal venoms, understanding the molecular mechanisms underlying variation in venom phenotypes is therefore crucial for clarifying their ecological functions and enhancing bioprospecting efforts [2].

Comparative studies of venom composition have become a central focus in venom evolution research and a promising avenue for bioprospection [9]. Such studies have revealed that venoms are complex secretions that contain a diverse mixture of bioactive compounds, including proteins, peptides, salts, and a range of organic and inorganic molecules [10,11]. In addition to proteins, small non-protein molecules such as alkaloids also contribute to the bioactivity of venom in some taxa [12,13,14].

The different molecules present in venom can target different physiological systems, producing diverse outcomes including neurotoxicity [15,16], cytotoxicity [17,18], hemotoxicity [19,20], and allergic reactions [21,22]. Isolated venom components may also be able to reproduce the effect of envenomation alone [23,24] or act synergistically to disrupt homeostasis. Such effects may arise from homologous toxins with shared evolutionary origins or from unrelated molecules that have convergently evolved to perform similar functions in envenomation [25]. This evolutionary versatility, coupled with the capacity to modulate multiple physiological systems in prey and predators, makes venoms a particularly rich source of novel bioactive molecules. Their extensive natural variation further enhances their biotechnological and pharmacological potential [26,27,28].

Venom variation also presents implications for drug biodiscovery and development of antivenoms. Geographical, ontogenetic and sex-specific differences in venom composition, driven by ecological, genetic and environmental factors, should be considered for the design of immunization mixtures and setting quality control criteria for effective antivenoms [4], as the effectiveness of these therapeutics relies heavily on the diversity and relative abundance of toxins present in the venom pool used for immunization [4,29]. Differences in venom composition have already been shown to reduce the efficiency of marketed antivenoms [30,31,32].

Understanding the sources of venom diversification is therefore as important as understanding their biochemical activities. At the molecular level, venom phenotypes are modified by a combination of point mutations in toxin-encoding genes, changes in auxiliary venom proteins, and alterations in regulatory networks operating at transcriptional, post-transcriptional and post-translational levels [33]. Although all these mechanisms are important and act together to shape the diversity of venom, historically venom research has placed greater emphasis on understanding the evolution of venoms through the impact of mutations within toxin-encoding genes [34,35]. As a consequence, less focus has been directed toward understanding the role of changes in regulatory elements within animal venoms or integrative approaches, combining different sources of data. This historical bias largely reflects the limited scope and higher costs of earlier methods, which constrained the systematic study of contributors at the regulatory and protein level to venom phenotypes.

In recent decades, however, advances in high-throughput sequencing technologies, along with decreasing financial and time costs, have transformed scientific research [36]. Such technological advances have allowed the exploration of venom and venom-associated tissues beyond the identification of toxin-related genes, enabling the comprehensive investigation of other molecular components, including genes with regulatory roles [37]. Specifically, omics technologies and sophisticated bioinformatics methods have facilitated detailed analyses of gene content and expression at multiple levels, including genomics [38], transcriptomics [39,40,41], and proteomics [40,42]. These efforts have provided important knowledge on the dynamics and evolutionary origins of venom systems, allowing comparisons between different tissues [43], populations [44], ontogenetic stages [45,46,47], and sex-specific differences in the same species [48,49]. This knowledge has been crucial for the identification of putative molecules and biological pathways or tissues that might be evolutionarily related to the origin of venom systems [37,43,50,51]. Moreover, comparative omics studies conducted across different time scales and a wide range of species can shed light on the evolutionary trajectory of venom systems throughout the Animal Kingdom [3].

In this way, a deeper understanding of the mechanisms of gene regulation in the venom gland is paramount to advance venom research. In this article, we review the literature on molecular mechanisms behind venom diversity at different levels. To guide the reader through the review, we first synthesize the ecological and biomedical significance of venom diversity (this introduction), then move to evidence-based summaries of molecular mechanisms shaping venom phenotypes and conclude with the benefits of comparative omics research for expanding knowledge regarding venom diversity and implications for the development of new drugs and enhancing biodiscovery.

## 2. Different Processes Can Lead to Venom Variation

Various molecular mechanisms, acting independently or in combination, can drive venom variation. To complicate the scenario, many of these processes can produce similar phenotypic outcomes, making it challenging to disentangle and identify the specific mechanisms responsible, particularly across different evolutionary timescales. In addition, plastic responses to ecological pressures (diet, ontogeny, and environment) can mimic or mask genetic differences, further complicating inference. Key contributors to venom diversity include cis-trans changes in gene regulation, gene duplication, alternative splicing, point mutations, and Darwinian selection [4,52]. Because several mechanisms can converge on similar proteomic or functional outcomes, robust experimental designs must integrate genomic, transcriptomic, and proteomic evidence before assigning causality.

### 2.1. Gene Regulation

**Figure 1:**
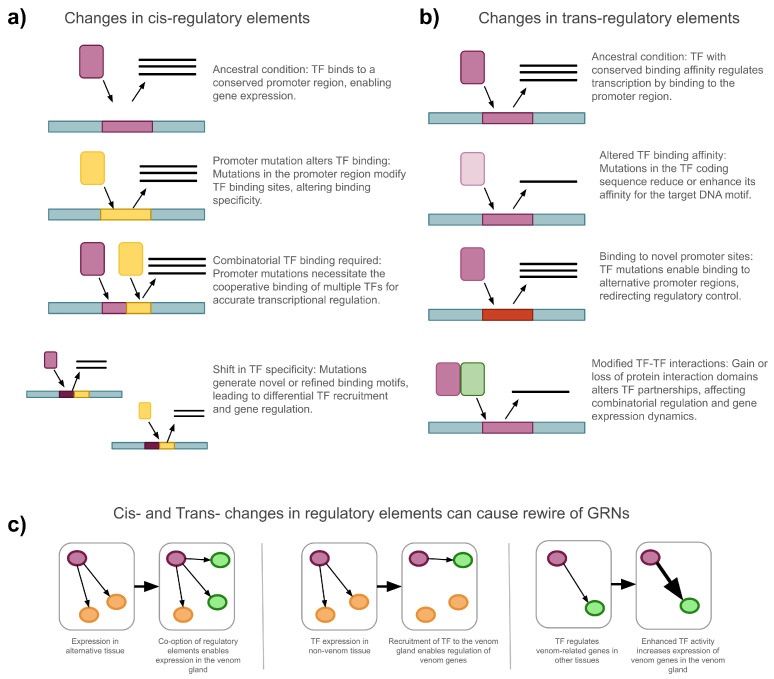
Examples of how cis- and trans-regulatory changes drive the evolution of GRNs underlying venom gene expression. (**a**) Changes in cis-regulatory elements: Mutations in promoter regions affect TF binding, leading to altered gene expression. Scenarios include changes in TF binding affinity due to single mutations, requirement for combinatorial TF binding, or the emergence of novel binding motifs that shift TF specificity. (**b**) Changes in trans-regulatory elements: Mutations in TF coding sequences alter TF binding affinity, enable binding to novel promoter sites, or modify protein–protein interactions between TFs, impacting regulatory dynamics and target gene expression. (**c**) Consequences of cis- and trans-changes for GRN rewiring: Stepwise modifications in regulatory elements and TFs enable the recruitment of genes into new expression contexts, such as the venom gland. This rewiring leads to co-option of genes, recruitment of TFs, and enhancement of venom gene expression through changes in regulatory architecture.

Gene regulation is a dynamic, multi-level process influenced by genetic mutations, epigenetic modifications, and environmental factors [53]. These influences can act at the chromatin, transcriptional, post-transcriptional, translational, and post-translational levels, shaping the spatial and temporal patterns of gene expression [54]. In an evolutionary context, such regulatory changes can result in significant phenotypic variation, driving the emergence, modification, or loss of traits [55]. The evolution of traits often involves the “rewiring” of Gene Regulatory Networks (GRNs), leading to changes in the hierarchical interactions between regulatory elements and their target genes. These changes in gene regulation are especially important, as these have been shown to play a more critical role in species adaptation and the origin of novel traits than changes in structural genes [56]. Furthermore, regulatory divergences are widely recognized as key drivers of species differentiation [57]. Multiple mechanisms can cause regulatory changes and are exemplified in Figure 1.

Changes in gene regulation, particularly in transcription factor (TF)–gene interactions, can be the outcome of modifications in either cis-acting or trans-acting elements. Cis-acting elements are non-coding DNA sequences, such as promoters, enhancers, and silencers, that regulate the expression of nearby genes [54]. Mutations in these regions can affect the binding affinity of TFs by altering sequence specificity, potentially creating binding sites that are more permissive, allowing interaction with multiple TFs, or more selective, limiting binding to specific TFs (Figure 1a). In contrast, trans-acting elements encode regulatory proteins, including TFs and transcriptional cofactors, which modulate gene expression by interacting with components of the transcriptional machinery to activate or repress transcription [55,58,59] (Figure 1b). Changes in both cis- and trans-acting elements can cause gene regulatory rewiring, affecting the molecular cascade of regulatory interactions (Figure 1c). The epigenomic state (chromatin accessibility, histone marks, DNA methylation) further conditions these interactions and provides evidence of changes throughout venom gland ontogeny [60].

The precise control of gene expression arises from the dynamic interplay between cis- and trans-acting elements, both of which evolve over time. This regulatory flexibility allows gene expression to respond to developmental cues and environmental conditions [53]. However, much remains unknown about how GRNs evolve and culminate in the expression of phenotypes. These processes are particularly relevant to understand venom evolution, where regulatory changes can cause the co-option of existing molecular pathways into venom systems [3,37,61]. Such shifts in expression patterns can drive the diversification of venom without requiring the evolution of entirely new genes.

One example of co-option of regulatory circuits is the upregulation of genes involved in protein folding within venom glands, particularly those regulated by the Unfolded Protein Response (UPR) and the Extracellular Signal-Regulated Kinase (ERK) pathways. TFs specific to the UPR (e.g., ATF6, BHLHA15, and XBP1), and those associated with the ERK pathway (e.g., EHF and GRHL1) were found to have putative binding sites in the promoter regions of venom-related genes [50,62]. Similar results were obtained for TFs (e.g., ATF4, ATF6B, CREB3L1, and CREB3L2) shared between both pathways, revealing the coordinated activation of these stress-response mechanisms in the evolution of venom systems [37,50,62]. These pathways likely support high secretory throughput by expanding protein-folding capacity and epithelial turnover. The positive-feedback model linking UPR/ERK activity to venom output has been presented as a working hypothesis that requires perturbation evidence for validation. In this model, the initial signs of venom synthesis trigger cellular stress, which activates these pathways. This in turn enhances the expression of venom genes [37].

The expression profile of the rattlesnake *Crotalus adamanteus* venom gland showed evidence for the role of TFs from MAPK/ERK signaling pathways as major regulators of the venom gland, and also unveiled a set of TFs that are differentially expressed between juvenile and adult life stages (enhanced in adults: Foxb2, Nr4a1, Etv1, Egr1, Snai3, Spi1, Nr4a3; enhanced in juveniles: Barx2, Sox8, Tfap2b, Klf15) [60]. In addition, epigenomic changes were identified in cis-regulatory elements located near venom genes in the comparison between juveniles and adults. The authors suggest that these regulatory modifications and the resulting shifts in venom composition may be associated with ontogenetic dietary changes observed between life stages [60]. Consistent with this, TF expression differences within and between different species of *Crotalus* further suggest fine-scale regulatory diversity as a driver of venom variation [61].

The upregulation of the UPR and ERK pathways in venom glands was also reported in a study by Zancolli et al. (2022), which aimed to identify shared gene regulatory mechanisms across venom systems with independent evolutionary origins [3]. Their comparative analysis included samples from wasps, spiders, scorpions, mollusks, a fish, a mammal, and snakes, and provided evidence of convergent evolution of co-option of molecular processes involved in maintaining venom gland function and stability, including UPR TFs (ATF6, PERK, IRE1), acting in response to secretory stress and venom gland cell turnover [3]. Further support for the co-option of stress-response pathways comes from a separate study by Kowalski et al. (2024), which focused on venomous shrews. Their analysis of venom gland extracts from two species (*Neomys fodiens* and *Sorex araneus*) revealed a high abundance of proteins involved in cellular stress responses and epithelial turnover [63].

Genes related to metabolic processes are also found to be upregulated in venom glands. Zancolli et al. (2022) demonstrated that venom glands exhibit expression profiles similar to other glandular tissues, indicating the recruitment of common glandular pathways for venom production [3], including pathways involved in amino acid, carbohydrate, lipid and fatty acid metabolism. Hogan et al. (2024) [60] reinforced support for the recruitment of stress-associated pathways for the stability and maintenance of the venom gland apparatuses in *Crotalus adamanteus*, while ontogenetic shifts required upregulation of molecular pathways associated with the circadian rhythm and biological timing systems in adults, followed by corresponding epigenomic alterations. The authors were also able to identify a set of TFs upregulated in the adult in comparison to young snakes, which might be associated with the respective phenotypic changes observed in venom composition [60].

In the ant *Tetramorium bicarinatum*, high TF activity was observed in the venom gland, representing a class of highly expressed genes, second only to toxins. Various TFs were found to be upregulated in the venom gland, including GATA TFs (e.g., GATA-Srp and GATA-Pnr) involved in the expression of secretory pathways [51]. Moreover, DNA promoter regions characteristic of GATA TF binding sites were observed for different venom genes [51]. In *Drosophila*, these TFs act in fat body development and in the regulation of defense-related peptides, suggesting convergent recruitment of GATA TFs to venom regulation in *T. bicarinatum* [32,51]. TFs homologous to fat body regulators also showed strong interactions with the toxin gene CRISP-3 in spiders [43], revealing a possible example of convergent evolution in the recruitment of GATA TFs for the venom apparatuses.

The formation of venom apparatuses in the cnidarian *Nematostella vectensis* reveals that specialized regulatory mechanisms underpin the development of venom systems from neural precursors [64]. The loss of NvPOU4, a member of the POU (Pit-Oct-Unc) family of TFs, results in the inability of neuronal cell precursors to undergo terminal differentiation, thereby inhibiting the formation of cnidocytes and other mature neuronal cell types [64]. Additionally, homologous TFs involved in the UPR pathway, known as Cnido-JUN and Cnido-FOS, were found to be upregulated within cnidocytes. However, these genes may have been recruited to cnidocytes for functions beyond stress response, as knockdown of Cnido-JUN leads to defective morphology of cnidocytes [65]. Modifications in the tbx4 developmental TF cluster also correlate with the appearance of toxic fins in fishes [66].

Furthermore, understanding the evolution of gene regulation in venom glands is critical to resolving a long-standing conundrum in the field: distinguishing proteins dedicated to envenomation from those with standard physiological roles, given that both belong to the same constitutive superfamilies and are expressed in the venom gland. Classical examples include enzymes and other proteins, as well as steroids [12,13,14,67]. One approach involves multi-tissue differential expression, isoform-level analysis and comparison of the gene regulation cascade of the same species relative to venom gland [3,43], coupled with careful functional annotation to trace orthologous/paralogous histories. Broad tissue and phylogenetic sampling has, for example, challenged the single-origin Toxicoferan venom hypothesis, since genes once considered venom-specific were also found to be expressed in non-venom tissues such as skin [68].

### 2.2. MicroRNAs

MicroRNAs (miRNAs) are non-protein-coding small regulatory RNA molecules, typically ranging from 21 to 25 nucleotides in length. miRNAs play a key role in post-transcriptional gene regulation and miRNA regulation affects at least one third of the protein-coding genes [69]. miRNA regulation occurs as described: miRNAs bind Argonaute (AGO) proteins to form the RNA-induced silencing complex (RISC) to guide this interaction. Once bound, they can either induce mRNA degradation or inhibit translation, a process collectively known as RNA interference (RNAi) [70]. miRNAs participate in an exceptionally broad spectrum of biological processes, ranging from the control of developmental timing and cell fate decisions to the fine-tuning of tissue homeostasis, with processes including cell differentiation, metabolism, and stress and immunity responses, reflecting a broad influence across metazoan physiology [71,72,73]. While studies of miRNA profiles (miRNAomes) in venomous animals are more scarce, these studies have revealed important roles of the miRNAome in development, evolution and, in some cases, a bona fide role as venom constituents is also suggested [45,46,71,74].

Venom composition alterations mediated by miRNA modulation were observed in rattlesnakes of the genus *Crotalus* [45,46]. Regulatory differences were investigated across three species (*C. simus*, *C. tzabcan*, and *C. culminatus*) at different ontogenetic stages (juveniles and adults) [46]. Several miRNAs whose abundance shifts were associated with increases in snake venom metalloproteinases (SVMPs) expression and concurrent repression of crotoxin were identified, supporting a regulatory switch that likely plays a relevant role in the ontogenetic transition observed in *C. simus*, where juvenile venom, characterized as type II (high ${\mathrm{PLA}}_{2}$ content), shifts toward a type I venom profile in adults, dominated by SVMPs [45,46]. However, this clear dichotomy between type I and type II venom was not observed in the other *Crotalus* species analyzed. In *C. tzabcan*, adult venom exhibited a reduction in crotamine content and an increase in SVMPs, crotoxin A and B, and C-type lectins (CTLs). In *C. culminatus*, both juvenile and adult venoms were characterized by high SVMP concentrations, with crotoxins absent in all developmental stages. Additionally, variation in the miRNAome was detected among allopatric populations within the same species, suggesting that microRNA-mediated regulation contributes not only to ontogenetic shifts but also to population-level venom diversity [46].

The comparison of miRNA expression in two snakes (one viperid and one elapid) before and after venom milking has revealed distinct miRNA profiles between the analyzed species. In the elapid *Pseudonaja textilis*, around 50% of the identified miRNAs were present in both stages compared (unmilked and milked), while the number of common miRNAs expressed in the viperid *Crotalus viridis* in comparison to *P. textilis* was only 18%. In *P. textilis*, a higher number of putative targets of miRNAs were found among venom genes: In *P. textilis*, C-type lectin-like toxins, ${\mathrm{PLA}}_{2}$s, pseutarin C catalytic subunit and non-catalytic subunit and 3FTxs were found as putative targets, and in *C. viridis*, two SVMP transcripts were found as putative targets [75]. These findings suggest that miRNA regulation may play a role in shaping venom composition, with potential differences in regulatory strategies between elapids and viperids [75], while reinforcing the regulation of SVMPs in viperids [46].

miRNA modulation can also have important impacts on the evolution of venom molecular systems. The miRNAome of the king cobra (*Ophiophagus hannah*) shows similarities to known profiles of human pancreas, suggesting the co-option of core pancreatic genetic regulatory components to venom systems [76]. While such similarity is correlative, it supports the broader idea that conserved secretory programs can be redeployed in venom glands. For cnidarians, the presence of a pan-cnidarian microRNA, miR-2022, was found to be fundamental for the biogenesis of cnidocytes, the stinging cells that contain nematocysts (cnidocyst), the venom-injecting organelles [77].

miRNAs might also act as part of the venom. The injection of a miRNA obtained from the venom of the naja *Naja atra*, CV-exo-miR-2904, in mice tail was followed by an increase in body temperature, decreased weight gain, and abnormal serum biochemistry, resulting in effects similar to cobra envenomation. Injection of CV-exo-miR-2904 into the liver of mice also led to edema and apoptosis of the tissue, again mimicking the effects of cobra envenomation. These experiments support the notion that this miRNA could be an actual part of the venom of the naja venom, rather than solely an endogenous regulator [74].

The role of miRNAs in toxinology has been primarily investigated in the context of the differential regulation caused in the prey/target during envenomation or in response to isolated venom components [78,79,80], rather than through direct examination of their mechanistic roles in envenomation. Progress in this area is hindered by several computational challenges, including the limited availability of representative miRNA sequence databases for non-model organisms. Moreover, miRNA target prediction often suffers from high false-positive rates and strong dependence on cellular and tissue context, complicating the establishment of causal links between miRNAs and their RNA targets. To overcome these limitations, future studies could benefit from sequencing approaches adapted from crosslinking and immunoprecipitation (CLIP) methods. In particular, Argonaute-CLIP (AGO-CLIP) offers a promising strategy for the experimental validation of miRNA–mRNA interactions [81,82]. Such approaches may help distinguish regulatory correlations from direct mechanistic interactions, and provide insights into whether miRNAs function primarily as gland-intrinsic regulators or as secreted components of venom.

### 2.3. Alternative Splicing

Alternative splicing (AS) is a post-transcriptional process that allows a single pre-mRNA to give rise to multiple mRNA isoforms through differential selection of splice sites. In the canonical splicing pathway, introns are removed and exons are joined in their genomic order, producing a single predominant mRNA isoform. By contrast, AS introduces variability by altering exon inclusion or splice site choice, thereby expanding transcript and consequently, proteome complexity [83,84].

AS is regulated by a complex interplay between cis-acting elements (e.g., exonic and intronic splicing enhancers and silencers) and trans-acting factors (serine/arginine-rich (SR) proteins and heterogeneous nuclear ribonucleoproteins (hnRNPs)). Together, these components determine splice site selection and influence exon inclusion or skipping [84,85]. At the core of this process is the spliceosome, a large ribonucleoprotein complex that recognizes consensus sequences at exon-intron boundaries and catalyzes intron removal followed by exon ligation [86]. Because individual transcripts often harbor multiple splice sites of varying signal strengths, spliceosome recognition may occur directly at strong sites or require the assistance of auxiliary regulatory proteins to ensure proper exon or intron retention or exclusion [86]. The principal AS modes include exon skipping (cassette exons), mutually exclusive exons, alternative $5^{\prime }$ and $3^{\prime }$ splice sites, and intron retention, each altering coding sequence and/or UTR structure with consequences for domain architecture, secretion signals, and RNA/protein stability [83,84].

In venom systems, AS can remodel signal peptides, propeptides, domain architectures, or disulfide frameworks, generating toxin variants with distinct stability, secretion, or target interactions, contributing to the complexity and adaptability of venom arsenal. In the Okinawa habu (*Protobothrops flavoviridis*), extensive occurrence of alternative splicing was observed for Metalloproteinases/Serine Peptidases and vascular endothelial growth factors. For Metalloproteinases specifically, the number of transcript variants was nearly eightfold higher than the 11 genomic loci identified [87]. In the stonefish *Synanceia verrucosa*, a large number of alternative splicing events were observed, with 411 isoforms generated from six neoVTX genes [88], a pore-forming toxin family associated with hemolytic and cardiotoxic activities [89,90].

In scorpions, venom peptide diversity is also influenced by alternative splicing. In *Lychas mucronatus*, LmTxLP11 and LmVP1.1 transcripts share an identical $5^{\prime }$ region and arise from a single locus via alternative splicing. LmVP1.1 probably has antimicrobial activity, whereas LmTxLP11 probably exihibits both antimicrobial and neurotoxic activities [91]. For *Mesobuthus martensii*, alternative splicing was observed in a protein related with $K^{+}$ channel modulation (BmKcug1 and BmKcug2), differing at their $3^{\prime }$-acceptor sites; this arrangement can confer greater stability to BmKcug2 and support efficient translation [92].

In a broader scope, Ye et al. (2023) reported that more than half of venom-associated genes in the parasitoid wasp *Pteromalus puparum* produce multiple isoforms within the venom gland, underscoring AS as a substantial source of toxin diversity in the group [93]. Similarly, a multi-omics study of *Parasteatoda tepidariorum* found that the number of transcript variants per gene is higher for genes contributing to venom than for non-venom genes [94].

High-quality reference genomes combined with long-read transcriptome data (e.g., Iso-Seq) provide the most reliable resolution of full-length isoforms and accurate exon mapping [95]. In contrast, short-read assemblies (e.g., Illumina) can complicate transcript reconstruction, sometimes leading to inflated isoform counts or difficulties in distinguishing closely related paralogs. To establish isoform-specific functions, peptide evidence should be validated through proteomics approaches such as targeted Parallel Reaction Monitoring (PRM), Selected Reaction Monitoring (SRM), or Data-Independent Acquisition (DIA) and, where feasible, complemented with top-down approaches to capture intact proteoforms. Functional assays are then essential to link isoform diversity to envenomation phenotypes [96].

Moreover, despite the prominence of both gene duplication (discussed below) and AS research for elucidating venom evolution, their interplay remains largely unexplored [97]. Given the critical roles that both processes play in shaping the complexity and diversity of animal venoms, an integrative framework that couples isoform-aware transcriptomics and proteomics with gene family evolution will be essential to understand venom diversification at the genomic and functional levels.

### 2.4. Gene Duplication

Gene duplication increases biological complexity and facilitates the emergence of novel traits, providing raw material for evolutionary processes [98]. Gene duplicates can arise through several mechanisms, including unequal crossing-over during meiosis, retroposition, and large-scale chromosomal or whole-genome duplications [99]. Unequal crossing-over typically generates tandem arrays, leaving duplicated genes linked on the same chromosome. The unequal crossing-over can lead to duplication of a gene, a part of a gene or several genes [99]. Retroposition, in contrast, produces intronless copies at new loci when an mRNA molecule is reverse-transcribed into cDNA and integrated into the genome [100]. At the moment of gene duplication, the resulting gene copies are indistinguishable. However, they can rapidly diverge over time through mutations and other evolutionary processes [101]. In the case of Whole-Genome duplications, a rarer event, all genes are duplicated simultaneously, creating abundant raw material for evolutionary innovation.

The birth of duplicate genes is a common event, yet the probability of a duplicate gene becoming fixed within a population is low [101,102]. Consequently, the vast majority of gene duplicates are lost shortly after their formation. The fate of a duplicated gene is influenced by multiple factors, including population dynamics like genetic drift, the action of natural selection [103], and pre-duplication functional constraints on the ancestral gene [99]. For those duplicates that escape initial loss, relaxed selective pressure enables three primary evolutionary trajectories: (i) Non-functionalization: One paralog accumulates deleterious mutations and is eventually lost from the genome [104]. (ii) Subfunctionalization: The duplicated genes partition the ancestral functions, with each copy specializing in a distinct subset of the original role [105]. (iii) Neofunctionalization: One paralog acquires a novel function not present in the ancestral gene, a process that can drive evolutionary innovation [104,105,106].

Gene duplication is widely recognized as a common driver of evolution for venom components and, in recent decades, substantial evidence of venom genes duplication has been gathered [107,108,109]. Although the model of duplication and subsequent neofunctionalization, where genes duplicate and are recruited for new toxic functions, is widely cited as working hypothesis in venom evolution research, growing evidence suggests this might not be the prevalent evolutionary pathway of venom genes. Researchers argue that a process of duplication and restriction, wherein gene duplicates undergo specialization for a narrowed subset of ancestral functions specifically within the venom gland, often provides a more parsimonious explanation [110,111,112].

Gene duplication and recruitment to the venom gland propel the evolutionary arms race between venomous animals and their prey [52,76,113]. In the king cobra (*Ophiophagus hannah*), multi-omics data (genomics, transcriptomics and proteomics) has revealed extensive gene expansion events in major toxin gene families, including phospholipase $A_{2}$ (${\mathrm{PLA}}_{2}$), metalloproteinases, and kallikrein-like serine proteases, suggesting a possible adaptive response to prey defenses driven by evolutionary pressure [76]. Similarly, in ticks, the diversification of gene families involved in hematophagy, often facilitated by gene duplication events, may represent an adaptive mechanism to counteract host immune responses [114,115,116].

In fishes, gene duplication coupled with positive selection has been proposed as a source of the evolution of ICTx protein-conding genes that became toxins, with recruitment to cells surrounding the venomous spines of these animals [117]. Conotoxins were also shown to be under strong regimes of gene duplication and rapid divergence of paralogous genes, with higher rates of gene duplication than observed for other multi-gene families with non-toxic-related functions. This might be associated with strong selective pressures, resulting from an evolutionary arms race between the toxins of *Conus* mollusks and prey ion-channel targets [118,119]. By contrast, the transcriptomics analysis of three widow spider species found no evidence of elevated rates of gene duplication and retention among genes with venom gland-biased expression genes, suggesting that gene duplication is common but may not be a predominant and universal mechanism across venomous taxa [120].

Whole genome duplications (WGDs) are rarer events but have also been documented across several taxa that include venomous organisms, including fishes and arachnids [120]. In ray-finned fishes, a potential positive correlation between successive rounds of genome duplication and species richness can be observed [121,122]. Similarly, successive whole-genome duplication events may have played a key role in the diversification of Arachnida, a clade that includes spiders, ticks and scorpions. Genomic evidence supports multiple genome duplication events along the evolutionary lineages leading to scorpions and spiders [123,124,125]. However, how these duplicates are incorporated into regulatory systems, has not been thoroughly investigated and remains an open question. Elucidating these events will require integrating comparative genomics with regulatory assays in venom gland tissues (e.g., chromatin accessibility and TF mapping), and further multi-omics analysis to resolve the individual role of duplicated genes in envenomation and venom gland stability.

The increasing availability of high-quality genomes, particularly those generated with long-read sequencing technologies, is significantly enhancing our ability to detect and analyze gene duplication events [38,126]. This technological progress provides an unprecedented opportunity to understand the evolutionary dynamics of how genes are recruited into the venom gland and incorporated into its regulatory networks. However, a major obstacle remains: the accurate identification of gene duplicates in complex genomic regions with high sequence similarity. To overcome this challenge, a combination of comparative genomics and functional assays are essential for elucidating the precise mechanisms driving venom evolution and adaptation.

### 2.5. Mutations and Amino Acid Substitutions

Mutations arise from DNA replication errors, recombination or due to DNA damage. Mutations can affect either cis-regulatory regions or protein-coding sequences [127], therefore holding potential implications for venom gene expression. Point mutations in protein-coding genes can be classified into synonymous (silent) or non-synonymous (missense or nonsense) mutations. Synonymous mutations have this name because they do not change the animo acid sequence of the encoded protein, due to the redundancy of the genetic code. However, these can still cause alterations in the expression of certain genes, altering the stability of the mRNA, its secondary structure, and the efficiency of translation [127]. Non-synonymous mutations, on the other hand, lead to changes in the amino acid sequence of the encoded protein, which can have direct effects on protein structure and function [128].

Mutations provide the raw material on which natural selection can act upon. Tests are conducted using the ratio of non-synonymous ($d_{N}$) substitutions over synonymous ($d_{S}$) substitutions ($d_{N}/d_{S}$) [129] in order to identify the selective pressures acting on a gene. A high $\omega =d_{N}/d_{S}$ indicates positive selection, while a lower rate suggests purifying (negative) or neutral selection. Therefore, analyzing the effects of mutations, whether or not they result in amino acid substitutions, is crucial not only for understanding structural and functional changes in proteins but also for uncovering the evolutionary forces shaping the evolution of genes. In practice, site, branch, and branch-site codon models are commonly used to localize selection in sequence alignments and polymorphism-aware tests (e.g., McDonald–Kreitman) can complement divergence-based inference [130,131,132].

Sunagar and Moran (2015) proposed a two-speed evolutionary model to explain the diversification of venom systems in animals, emphasizing the role of natural selection. According to this model, venoms in ancient lineages tend to evolve under strong purifying (negative) selection, maintaining functional stability. In contrast, toxin gene families in more recently diverged lineages exhibit rapid diversification driven by positive selection, often associated with periods of ecological niche expansion and specialization [28]. However, studies focused on more specific lineages and protein families have revealed contrasting patterns, even within closely related species, highlighting the complexity and lineage-specific nature of venom evolution [133]. The evolution rate in specific scorpion toxin families, considered an old lineage, also defies this hypothesis [134]. In *Hadrurus* scorpions, αKTX toxins that target voltage-gated channels were found to be under strong regimes of natural selection [134].

Rokyta et al. (2015), by comparing coding sequences from two *Crotalus* species (*C. horridus* and *C. adamanteus*), found a higher average of $d_{N}/d_{S}$ for toxin-genes compared with non-toxin genes [133]. The same pattern of evolutionary rates in toxin-related genes relative to constitutive genes was observed in species of the genus *Protobothrops* (*P. elegans* and *P. flavoviridis*), especially for highly abundant toxins, including ${\mathrm{PLA}}_{2}$, CTL, Metallopeptidases and Serinepeptidases [135,136]. However, the same was not observed for the Crotalinae *Cerrophidion godmani*, with non-toxin genes presenting a higher Tajima’s D (high polymorphism than expected under a neutral model), significantly different from 0, while toxin genes did not present a Tajima’s D significantly different from zero. The authors hypothesized that this might indicate that toxin genes are evolving through a mutation-drift scenario, or that the highly variable Tajima’s D values obtained for different toxins reflect a loss in the evolutionary signal [137].

Inference of positive selection from $d_{N}/d_{S}$ can be confounded by alignment error, hidden paralogy in multi-copy toxin families, recombination/gene conversion, multinucleotide mutations (which can mimic recurrent adaptive change), saturation at synonymous sites, and expression-level constraints. To overcome these caveats, some practices can be adopted, including: (i) careful codon-aware alignment and paralog curation; (ii) screening for recombination before selection tests; (iii) applying complementary site/branch-site models and polymorphism-based tests; and (iv) integrating sequence evidence with expression, proteomics, structural modeling, and functional assays to confirm phenotypic effects related to nucleotide mutations and amino acid substitutions [131,138,139].

### 2.6. Post-Translational Modifications

Post-translational modifications (PTMs) involve proteolytic cleavage or the addition of modifying groups (e.g., acetyl, phosphoryl, glycosyl) to one or more amino acids [140,141]. These modifications influence protein structure, stability, activity, and interactions with molecular targets [142,143]. PTMs are commonly found in secretory proteins [141], including venom components. Importantly, the diversity of PTMs can generate multiple proteoforms from a single gene, thereby increasing venom complexity through a strategy that complements, rather than replaces, the recruitment of diverse protein families [143,144].

The characterization of proteoforms is most effectively achieved through top-down proteomics, which directly analyzes intact proteins and the respective PTMs. This contrasts with bottom-up proteomics, where proteins are identified indirectly through their peptide fragments [140]. While bottom-up strategies remain widely used, they require careful selection of fragmentation methods that preserve PTM information during peptide dissociation, if this is the research goal. In contrast, top-down approaches allow the direct identification and quantification of proteoforms, including those carrying PTMs, thereby providing a more comprehensive view of venom complexity [144]. For instance, a combined top-down and bottom-up analysis of king cobra (*Ophiophagus hannah*) venom identified 184 proteoforms, with three-finger toxins (3FTxs) being the most extensively modified. Additional PTMs were also detected in ${\mathrm{PLA}}_{2}$s, Kunitz-type serine protease inhibitors, and CRISPs, while distinct LAAO proteoforms were differentiated based on their N-glycan profiles [145].

In viperid snakes, glycosylation is one of the most prominent PTM [146]. A comparative glycoproteomic analysis of seven *Bothrops* species, using lectin-affinity chromatography and mass spectrometry, revealed that venom glycosylation patterns closely mirror phylogenetic relationships among these taxa [146]. In this study, most glycosylated proteins were SVMPs and SVSPs. The authors also proposed that sialic acid-containing glycans on these toxins, which are similar to glycans found on many host cell receptors, may facilitate toxin–target interactions during envenomation. Other venom components, such as ${\mathrm{PLA}}_{2}$s and CTLs, also exhibit glycosylation.

Glycosylation has also been reported in arthropod venoms, including those of scorpions and wasps. The first glycoprotein neurotoxin described in scorpions was Aah VI, an anti-insect toxin from *Androctonus australis hector*; however, its functional dependence on glycosylation remains unresolved [147]. A peptidomic analysis of *Tityus serrulatus* venom identified 227 modified tryptic peptides, including 129 phosphopeptides, 95 N-linked glycopeptides, and 3 peptides carrying both modifications. Another example is Phaiodactylipin, a ${\mathrm{PLA}}_{2}$ protein from *Anuroctonus phaiodactylus*, which contains three glycosylation sites [143]. Despite these findings, the biological significance of glycosylation in scorpion venoms remains poorly understood.

In the social wasp *Polybia paulista*, shotgun proteomics identified 1673 proteins, among which 23 showed N-linked glycosylation [148]. Three molecular forms of the venom allergen ${\mathrm{PLA}}_{1}$ were reported, two of which carried N-linked glycans. Hyaluronidases, another major allergen in wasp venom, are also glycoproteins and play a critical role in cross-reactivity between wasp and bee venoms in allergic patients [149].

Disulfide bridges are another common PTM widely observed in venom proteins across diverse species, including snakes, spiders, scorpions, leeches, and marine snails [150]. Owing to the secretory nature of venoms, these covalent bonds enhance protein thermostability, protecting them from proteolytic degradation and denaturation [151]. Many conopeptides, for instance, are cysteine-rich and stabilized by two to four disulfide bridges [152]. Disulfide bonds have also been found to link venom heterodimers of 3FTxs in *Naja kaouthia*. The addition of a disulfide bridge in venom phospholipases D of Loxosceles spiders differentiates different classes of phospholipases D. Class II proteins are identified with two disulfide bonds in opposition to one disulfide bond in class I proteins [153,154].

Other PTMs also contribute to venom protein integrity and bioprospecting potential. For example, many scorpion venom peptides exhibit C-terminal amidation, which protects against carboxypeptidase degradation and thereby increases peptide stability and half-life [155]. This modification is also common in mammalian neuropeptides and hormones [155,156], suggesting a possible role in molecular target recognition [155]. In bee venoms, classical components such as icarapins and melittins carry phosphorylation sites that serve as allergenic epitopes [157,158]. These phosphorylation events may enhance local tissue damage and exacerbate inflammation and allergic reactions in higher organisms [157,159].

The diversity and complexity of PTMs highlights the current lack of comprehensive databases cataloging venom-specific modifications. Although general PTM repositories are available, they are strongly biased toward model organisms and thus fail to capture the distinctive PTM landscapes of venomous species. The application of top-down proteomics, which enables the direct analysis of intact proteins while preserving PTM information, is further limited by the requirement for highly specialized instrumentation. Additional challenges include the need for multidimensional separation, difficulties in analyzing large proteins (>30 kDa), and the complexity of proteoform data acquisition and processing [160]. Integrating top-down proteomics with genomic and transcriptomic datasets, however, provides a promising avenue to overcome these limitations [160,161].

## 3. Brief Conclusions

The vast literature on venom research reveals that venom is a highly complex system, shaped by several molecular mechanisms, acting alone or in combination, and in different time scales. Although the advances in sequencing technologies and data analysis have increased the power for more robust annotation of venom components and comparative approaches for understanding the evolutionary aspect and enhancing bioprospection, there are still many open questions and challenges to be addressed. For instance, the molecular mechanisms that trigger the addition of PTMs or alternative splicing events are still poorly understood. The integration of multiple data layers, including genomics, transcriptomics, proteomics, and functional assays, will be essential to unravel the complexity of venom systems and their evolutionary dynamics.

## 4. Comparative Omics as a Framework for Understanding Venom Diversification

In the last two decades, a revolution in the analysis of biological data arose from the development of high-throughput technologies. These advances have allowed the identification of thousands of molecules in a single experiment, at an unprecedented scale. This technological leap has been especially impactful for venomics, where analyses of venom composition and identification of venom components have accelerated dramatically. The application of high-throughput sequencing technologies, such as RNA-seq and mass spectrometry, has enabled researchers to explore the complexity of venom gene expression at unrivaled levels of detail [2]. The use of such methods, coupled with powerful computational tools for data analysis, has facilitated the identification of toxin and non-toxin genes, as well as the profiling of proteins, peptides, post-translationally modified proteoforms, and other bioactive compounds, providing valuable insights into the composition and function of venoms and other components.

Given the extensive number of molecules sequenced in a single experiment, omics approaches can aid in uncovering the relevant molecular processes responsible for toxin gene duplication, including the incorporation of duplicated genes to venom systems as well as the co-option of previously existing genes and pathways into the regulatory context of the venom gland [3,35,60]. This reinforces the necessity for broader comparative approaches at different levels to better understand the recruitment dynamics of venom systems [2]. For example, a recent comparative transcriptomics approach provided evidence supporting the hypothesis that venom glands and spider silk glands are evolutionarily closer due to more recent shared-ancestrality [43]. This conclusion was based on RNA-seq comparisons of various tissues, including silk and venom glands, as well as other glandular tissues. The study found a greater overlap among genes differentially expressed in the venom and silk glands than between venom and salivary glands, suggesting a more conserved core GRN between venom and silk glands. However, the absence of tissues previously identified as potential common origins with the venom gland, such as the coxal glands [162] and midgut [67], limits phylogenetic and developmental inferences [162]. Adopting a similar approach, Barua and Mikheyev (2021) found that the expression of the venom gland of *Protobothrops mucrosquamatus* was most similar to the expression of the salivary gland of other vertebrates compared to other tissues, suggesting an evolutionary link between venom and salivary glands [35].

The integration of multiple data layers provides a broader and more detailed perspective on venom systems. In particular, multi-omics approaches that combine genomics, transcriptomics, and proteomics have emerged as powerful strategies for uncovering the molecular mechanisms underlying venom production and the evolution of venom systems [3]. As cited in previous sections, comparative analyses of venom gland expression profiles across species, developmental stages, and environmental conditions have been able to uncover key genes, regulatory elements, isoforms, and proteoforms involved in venom production. Recent advances further enhance resolution: single-cell and spatial transcriptomics reveal cell type composition and reduce confounding from bulk tissue RNA-seq, while long-read sequencing enables the direct linkage of splice isoforms to full-length coding sequences. On the proteomics side, discovery approaches such as Data-Dependent Acquisition (DDA) and DIA, and targeted approaches, quantify toxins and validate isoform-specific peptides, whereas top-down proteomics directly characterizes proteoforms and PTMs that influence toxin activity and immunogenicity [163,164]. However, the analysis of such data requires specialized computational tools and pipelines, as well as careful experimental design to ensure robust and reproducible results [88,165].

### Best Practices for Data Acquisition and Analysis

The design of single-species and comparative genomics studies requires careful attention to both biological and technical factors to ensure robust and reproducible results. Standardization of animal handling, including venom extraction, tissue sampling, and storage, is critical to minimize variability and enable meaningful comparisons across samples and studies [165]. Equally important are study design decisions such as species and tissue selection, the number of biological replicates, and the choice of analytical methods. In transcriptomic studies, additional wet-lab variables, including tissue extraction procedures, RNA quality and integrity, library preparation protocols, and sequencing platforms, can introduce bias and must be carefully controlled. Similarly, in proteomic workflows, protein quantification and quality control, digestion protocols, peptide fractionation, mass spectrometry settings, and data acquisition strategies (e.g., DDA vs. DIA) directly affect the reliability of the results.

On the computational side, rigorous quality control and careful selection of algorithms and pipelines are essential at every stage of analysis, including read trimming, genome or transcriptome assembly, mapping, quantification, normalization, and differential expression analysis [88,165]. These practices form the foundation for generating high-quality, comparable omics datasets, which are critical for advancing comparative venom research. For example, a systematic evaluation of *de novo* transcriptome assembly methods demonstrated that assembler choice strongly influences assembly quality and downstream analyses, leading the authors to recommend the use of multiple assemblers to achieve more comprehensive and accurate transcriptomes [166]. This strategy has proven effective in practice: the use of multiple assemblers increased the recovery of theraphotoxins in the *Pamphobeteus verdolaga* transcriptome, underscoring its potential for enhancing bioprospecting efforts [167].

In the case of multiple samples and biological replicates, batch effects can arise from differences in sample processing, library preparation, and sequencing runs. These effects can confound biological signals and lead to false positives or negatives if not properly addressed. Statistical methods, such as surrogate variable analysis (SVA) and ComBat, can be used to identify and correct for batch effects in the data [168,169]. In the context of multi-species analysis, orthology-aware methods are essential for the accurate identification of orthologs, paralogs and further gene expression comparisons across species [170].

Such approaches increase the confidence in the results and enable more accurate biological interpretations. The integration of multiple omics layers, such as genomics, transcriptomics, and proteomics, can provide a more comprehensive understanding of venom systems. The adoption of such measures is pivotal for advancing venom research and bioprospection, ultimately leading to the discovery of novel venom components and therapeutic applications.

## 5. Venom-Derived Applications for Biotechnological Discovery

The remarkable diversity of animal venoms, shaped by distinct evolutionary pressures and molecular mechanisms, represents an exceptional source of bioactive compounds with therapeutic potential. Extensive variation in toxin structure, function, and molecular targets has generated a rich repertoire of molecules that can inspire or directly yield pharmacological agents. The successful approval of drugs derived from venom components further accentuates the importance of venom research for bioprospecting and drug discovery for the identification of promising drug leads [171,172]. At the same time, translation of venom-derived compounds into therapeutic applications requires careful consideration of developability factors (e.g., stability, delivery, immunogenicity) and on-target liabilities (e.g., bleeding risk for anti-platelet agents).

The development of anti-platelet aggregation agents derived from snake venom disintegrins represents a successful case of bioprospection, enabled by broad comparative screening of venom variation across species. Disintegrins are a class of low-molecular-weight, non-enzymatic proteins with potent antithrombotic activity, primarily through the inhibition of platelet aggregation. They are commonly found in venoms of various viperid snake species [173,174,175]. In 1988, a disintegrin named echistatin was isolated from the venom of *Echis carinatus*. Echistatin contains an arginine–glycine–aspartic acid (RGD) sequence that enables binding to multiple integrins, including αIIbβ3, αvβ3, αvβ5, and α5β1 integrins. Despite its efficacy, the lack of specificity toward αIIbβ3 limited echistatin’s therapeutic potential. To identify a more selective inhibitor, venoms from over 60 snake species were screened for disintegrins with the desired pharmacological profile. The most promising candidate was barbourin, isolated from the venom of *Sistrurus barbouri*. Barbourin exhibited high specificity for αIIbβ3, selectively inhibiting fibrinogen binding to the platelet glycoprotein IIb/IIIa receptor while sparing other RGD-recognizing integrins. This specificity was attributed to the presence of a lysine-glycine-aspartic acid (KGD) motif, which functionally replaces the canonical RGD sequence found in other disintegrins [176,177].

Exenatide represents another example of bioprospecting initiated from a molecule with known desirable biological activity. GLP-1 is an endogenous peptide in humans that regulates appetite and metabolism. During the investigation of *Heloderma suspectum* (*Gila monster*) saliva, researchers identified a homologous molecule, exendin-4, which shares 53% sequence similarity with human GLP-1. Exendin-4 and GLP-1 compete for the same receptor targets, eliciting similar physiological effects, including inducing satiety, reducing food intake, decreasing fat deposition, and promoting weight loss [178,179,180,181,182]. In addition, exendin-4 shows enhanced resistance to proteolysis (e.g., DPP-4 cleavage) and greater in vivo stability compared to human GLP-1, enhancing its suitability for drug development [182,183]. The development of exenatide thus highlights how bioprospecting can leverage molecular discoveries from both venomous and non-venomous organisms to identify and optimize molecules with therapeutic potential.

Other drugs have been developed based on molecular components present across a wide range of species, further underscoring venoms and other substances as versatile discovery spaces. For example, conotoxin peptides derived from the venom of *Conus* mollusks target diverse voltage-gated ion channels and receptors. A single *Conus* species can produce between 100 and 400 distinct peptides in its venom, and these peptides are among the fastest-evolving protein families identified to date [28,184]. One successful application of conotoxins is the development of the anesthetic ziconotide, a synthetic version of the conotoxin ω-MVIIA from *Conus magus* [185,186]. The conotoxin ω-MVIIA was first discovered through basic research on venom proteins from *C. magus* and *C. geographus*. The homologous peptide from *C. geographus*, known as ω-conotoxin GVIA, shares more than 70% sequence similarity with ω-MVIIA. However, their functional properties differ: while ω-conotoxin GVIA binds almost irreversibly to its target calcium channel, ω-MVIIA, despite being a high-affinity and highly selective ligand, can be washed out after binding [186].

In addition to the well-established relevance in the development of human therapeutics, venom-derived molecules also represent a valuable resource for the generation of other venom-based biotechnological products, including biopesticides [187]. Given the ecological roles of many venomous animals as insect predators and parasitoids, their venoms have evolved under strong selective pressures within an evolutionary arms race. As a result, these venoms constitute a promising reservoir for the discovery of novel bioinsecticidal compounds and the development of insect-resistant crop varieties [188]. Many venom components exert neurotoxic effects, frequently targeting ion channels in the nervous system [187], a mechanism particularly effective for insect control. Among venomous taxa, hymenopterans offer a rich source for the bioprospection of venom-derived bioactive molecules. For instance, poneritoxins (α-helical ponericins) from various *Neoponera* ant species have demonstrated diverse biological activities [189]. The peptide M-PONTX-Ng3a, isolated from *N. goeldii*, exhibits both insecticidal activity against cricket larvae and antimicrobial properties [189,190]. Similarly, M-PONTX-Nc3a from *N. commutata* has shown toxic activity against adult blowflies (*Lucilia cuprina*). M-PONTX-Na1b from *N. apicalis* venom has been characterized as an anthelmintic peptide active against parasitic nematodes [189,191].

The relevance of venom-based research for drug discovery is more than established, with rich examples present in the literature. Moreover, advances in omics technologies, combined with the establishment of large databases compiling venom-derived sequences, have created important resources for the initial in silico prospection of promising candidates, including the search for similar molecules already described [192]. The development of sophisticated bioinformatics methods for the in vivo refinement of potential molecules with desirable physiological activities further holds significant promise for accelerating the entire drug development pipeline by reducing the pool of initial candidates. Such workflows typically integrate three-dimensional structural modeling, evaluation of binding affinity between candidates and targets [193], and molecular dynamics simulations to assess the stability and interaction dynamics of the complexes [194,195].

The field of in vivo experimentation and prediction is undergoing a major transformation, driven by advances in artificial intelligence. In venom research, deep learning has enabled the de novo design of proteins targeting short- and long-chain α-neurotoxins from the 3FTX family. These designed proteins exhibited high thermostability and strong binding affinity to the toxins, and in vitro assays demonstrated their ability to neutralize multiple 3FTX subfamilies. Importantly, in vivo experiments showed protection against neurotoxic lethal effects in mice [196].

A broader approach was taken by Guan et al. (2025), who computationally screened thousands of venom proteins and generated millions of peptides, ultimately identifying 386 candidates with predicted antimicrobial activity distinct from known antimicrobial peptides. From these, 58 peptides were selected for in vitro validation, of which 53 displayed antimicrobial potential consistent with AMP-like mechanisms. One peptide was further tested in a murine model of *Acinetobacter baumannii* skin infection, where it significantly reduced bacterial load without detectable toxicity [197].

The application of such strategies is not only cost-effective but also reduces the need for extensive laboratory experiments during the early stages of drug discovery. By prioritizing candidates with the highest predicted efficacy and safety profiles, these computational approaches streamline the drug development process, ultimately accelerating the translation of venom-derived molecules into clinically viable therapeutics. Moreover, similar strategies can also be applied to the rational design of improved antivenoms with broader or specific scope [198].

## 6. Conclusions

Animal venoms constitute an important asset for the discovery of novel bioactive molecules with desirable properties. These are important drivers of biotechnological innovation, serving as research basis across multiple fields. Moreover, the extensive variation observed in venoms, across several levels, further enhances their potential for biorospection. Therefore, understanding the molecular basis underlying the variation in venom is crucial for the rational prioritization of venomous species in bioprospecting efforts. Although experimental, financial, and computational limitations have hindered in-depth studies on the molecular basis behind the expression of venom phenotypes, the on-going multi-omics revolution, along with the development of bioinformatics approaches, promises to greatly enhance future research. Moreover, the in-depth study of the mechanistic basis behind venom phenotypes will elucidate the evolutionary processes shaping venom systems. Given the extensive number of venomous lineages, understanding the molecular convergence of mechanisms that led to the rise of venom systems, as well as the diversification of venom components, will provide important knowledge regarding the evolutionary dynamics of such a fascinating trait.

## Data Availability

No new data were created or analyzed in this study.
